# Association Between Sodium–Glucose Cotransporter‐2 Inhibitors and Sepsis Risk in Patients With Type 2 Diabetes Mellitus

**DOI:** 10.1155/jdr/1437417

**Published:** 2026-01-08

**Authors:** Chun-Ying Wu, Chih-Cheng Lai, Chung-Han Ho, Yu-Cih Wu, Kuang-Ming Liao, Jhi-Joung Wang, Ding-Chau Wang, Fu-Wen Liang

**Affiliations:** ^1^ Department of Internal Medicine, Chi Mei Medical Center, Tainan, Taiwan, chimei.org.tw; ^2^ Division of Hospital Medicine, Department of Internal Medicine, Chi Mei Medical Center, Tainan, Taiwan, chimei.org.tw; ^3^ School of Medicine, College of Medicine, National Sun Yat-sen University, Kaohsiung, Taiwan, nsysu.edu.tw; ^4^ Department of Medical Research, Chi Mei Medical Center, Tainan, Taiwan, chimei.org.tw; ^5^ Department of Information Management, Southern Taiwan University of Science and Technology, Tainan, Taiwan, stust.edu.tw; ^6^ Department of Internal Medicine, Chi Mei Medical Center, Tainan, Taiwan, chimei.org.tw; ^7^ Department of Nursing, Min-Hwei Junior College of Health Care Management, Tainan, Taiwan, mhchcm.edu.tw; ^8^ Department of Public Health, College of Health Sciences, Kaohsiung Medical University, Kaohsiung, Taiwan, kmu.edu.tw; ^9^ Department of Medical Research, Kaohsiung Medical University Hospital, Kaohsiung, Taiwan, kmuh.org.tw; ^10^ Center for Big Data Research, Kaohsiung Medical University, Kaohsiung, Taiwan, kmu.edu.tw

**Keywords:** DPP4, NHIRD, sepsis, septic shock, SGLT2, type 2 diabetes mellitus

## Abstract

**Aims:**

This retrospective study is aimed at evaluating the effect of SGLT2 inhibitors (SGLT2i) on the risk of sepsis among patients with Type 2 diabetes mellitus (T2DM).

**Materials and Methods:**

An active‐comparator new user design was utilized based on Taiwan′s National Health Insurance Research Database. Adult patients diagnosed with T2DM between 2017 and 2020 who initiated either SGLT2i or DPP‐4 inhibitors (DPP‐4i) were included. After 1:2 matching by age, sex, and Charlson Comorbidity Index, the study enrolled 34,672 SGLT2i users and 69,344 DPP‐4i users. The primary outcome was sepsis, with secondary outcomes including septic shock, organ dysfunction, and mortality. Hazard ratios (HRs) were estimated using Cox proportional hazards regression.

**Results:**

SGLT2i users had a significantly lower risk of sepsis than DPP‐4i users (10.1% vs. 15.5%; adjusted HR [aHR]: 0.77; 95% CI: 0.74–0.81). The risk of septic shock and mortality in SGLT2i users was also reduced by 30% (aHR: 0.70; 95% CI: 0.57–0.87) and 40% (aHR: 0.60; 95% CI: 0.54–0.66), respectively, compared with that of DPP‐4i users.

**Conclusions:**

The findings suggest that SGLT2i is associated with a significantly lower risk of sepsis, septic shock, and mortality compared with that of DPP‐4i in patients with T2DM.

## 1. Introduction

Diabetes Mellitus (DM) is a common chronic disease and a global public health challenge. The estimated global mean trend of DM incidence from 1990 to 2019 showed an upward trajectory, with an annual increase of 3.73 cases per 100,000 people [1]. This rising incidence of DM was observed in both developing and developed countries [1]. Although both DM mortality and mortality‐to‐incidence ratio have shown a decline since 2005, with annual decreases of 0.14 and 0.001 per 100,000 people, respectively [1], DM remains associated with numerous adverse effects, including microvascular (nephropathy, retinopathy, and neuropathy) and macrovascular (myocardial infarction and stroke) complications [2–4]. In addition, DM can alter innate immunity and impair immune function [5], increasing susceptibility to infections such as COVID‐19, influenza, mucormycosis, pneumonia, intra‐abdominal infections, genitourinary infections, and skin and soft tissue infections, particularly in patients with poorly controlled DM [6–9]. Patients with diabetes often experience more severe infections, higher rates of acute kidney injury, and complications such as the need for ventilator support and vasopressors during episodes of sepsis, all of which are associated with increased mortality [10, 11]. In addition, chronic hyperglycemia and metabolic disturbances related to diabetes exacerbate the host response to infection, leading to persistent immune dysfunction even after the acute phase of sepsis has resolved. This contributes to long‐term morbidity and an elevated risk of recurrent infections [11, 12]. Given that DM is a major risk factor for poor outcomes in sepsis and septic shock, preventing sepsis in this population has become a critical clinical priority.

Recently, sodium–glucose cotransporter 2 inhibitors (SGLT2is) have emerged as a significant advancement in the management of Type 2 diabetes mellitus (T2DM). In addition to glycemic control, SGLT2is have demonstrated multiple beneficial effects. Notably, large‐scale clinical trials have shown that SGLT2is significantly reduce the risk of major adverse cardiovascular events, hospitalization for heart failure, and progression of diabetic kidney disease [13–17]. The combination of improved glycemic control and these additional benefits has positioned SGLT2i as a valuable therapeutic option in the comprehensive management of T2DM, particularly in patients with or at high risk of cardiovascular and renal complications.

Beyond these advantages, SGLT2is also exert pleiotropic and anti‐inflammatory effects, potentially reducing the risk of infections [18]. A meta‐analysis of 26 randomized, placebo‐controlled trials involving 59,264 patients demonstrated that SGLT2is significantly reduced the risk of pneumonia (pooled relative risk [RR], 0.87; 95% confidence interval [95% CI], 0.78–0.98) and septic shock (pooled RR, 0.65; 95% CI, 0.44–0.95) [19]. However, ascertainment bias remains a concern, as pneumonia and septic shock were not prespecified outcomes in the included trials. Moreover, real‐world evidence regarding the association between SGLT2i use and sepsis risk is limited [20]. Therefore, this retrospective study was conducted to assess the effect of SGLT2is on the risk of sepsis among patients with T2DM. An active‐comparator new‐user (ACNU) design was employed to enhance the validity and applicability of the findings by promoting group comparability, reducing confounding, and addressing clinically relevant treatment decisions [21]. Dipeptidyl peptidase‐4 inhibitors (DPP‐4is) were selected as the comparator group, as both SGLT2 inhibitors and DPP‐4 inhibitors are newer classes of oral antidiabetic agents and are typically prescribed to patients at similar stages of T2DM management [22].

## 2. Materials and Methods

### 2.1. Data Source

In this study, Taiwan′s National Health Insurance Research Database (NHIRD) was used to identify DM patients with the related comorbidities and those prescribed SGLT2i and DPP‐4i. The NHIRD was established according to the National Health Insurance of Taiwan, which is a nationwide health care program and enrolled more than 99.6% of the population. The database included detailed information regarding diagnostic codes, date of diagnosis, payments for consultations, and prescription details. The diagnosis codes in NHIRD were based on the International Classification of Diseases, 10th Revision, and Clinical Modification (ICD‐10‐CM) codes for diagnoses and procedures. Moreover, drug prescriptions were defined by the Anatomical Therapeutic Chemical (ATC) classification codes. With almost 100% of Taiwan′s population registered in the NHIRD, this database can show the evidence to support clinical decision‐making and health policy development [23].

### 2.2. Study Participants

In this study, T2DM was identified using the ICD‐10‐CM code: E11. All adult patients with T2DM diagnosis between 2017 and 2020 were selected from NHIRD. For reducing the potential confounding and selection bias, T2DM patients with a diagnosis of T2DM with SGLT2i (ATC code: A10BK) or DPP‐4i (ATC code: A10BH) were identified as qualified study subjects according to ACNU design [21]. An ACNU design was used to improve internal validity by reducing both confounding by indication and selection bias. In this study, new users of SGLT2is were compared to new users of DPP‐4is, an alternative glucose‐lowering therapy with similar clinical indications. This approach increased comparability by ensuring that both groups received initial treatment for the same underlying condition at a similar stage of disease progression [21, 24, 25]. Moreover, the ACNU design could reduce potential immortal time bias for better reflecting real‐world clinical decision‐making [25, 26].

**Figure 1:**
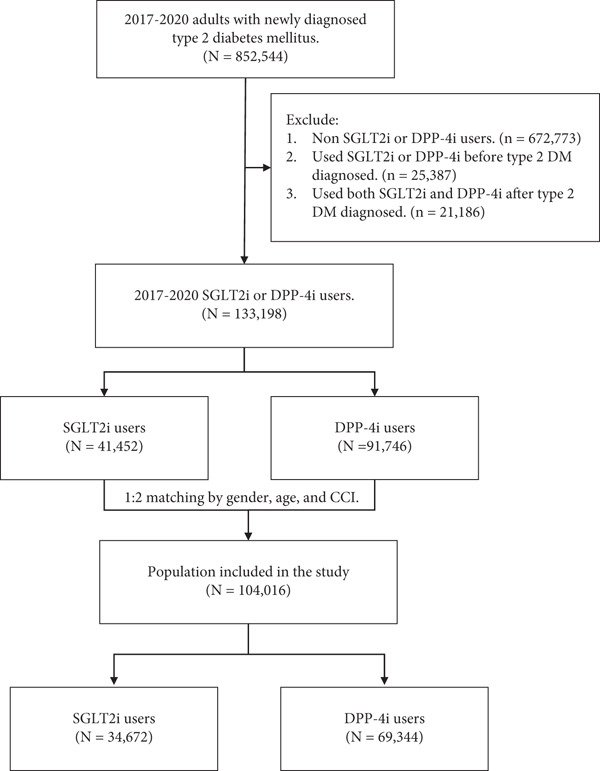
Flowchart of study subjects′ selection.

Case cohorts were defined as T2DM patients who used SGLT2i after DM diagnosis. In addition, the comparison cohorts were selected from T2DM patients who used DPP‐4i during the same period. New users were defined as patients with no prescriptions for either SGLT2i or DPP‐4i during the 365 days prior to the index date, ensuring a one‐year washout period. The index date was defined as the date of the first SGLT2i or DPP‐4i prescription. T2DM patients who were taking both SGLT2i and DPP‐4i during the study period were excluded to avoid potential drug–drug interactions. For minimizing the confounding effects, an exact matching approach was applied at a 1:2 ratio, matching each SGLT2i user with two DPP‐4i users based on sex, age group, and Charlson Comorbidity Index (CCI) category, without applying any tolerance margin. The CCI score was calculated using diagnostic records from the 12 months prior to the index date. All study subjects were right‐censored to follow until a first diagnosis of sepsis, death, or the end date of study date, December 31, 2021. Considering treatment adherence and the potential for switching medications, an intention‐to‐treat (ITT) principle was applied, in which exposure was defined based on the initial treatment assignment. This approach may better reflect real‐world clinical practice and ensure comparability of initial treatment strategies under the ACNU design. The selection of study subjects is illustrated as a flow diagram in Figure 1.

### 2.3. Outcome and Measurements

The primary aim of this study was to estimate the effect of SGLT2i for the risk of sepsis among T2DM patients. Study subjects developed sepsis using ICD‐10‐CM coding (Table S1) to define sepsis based on the previous studies [27, 28]. To further explore the impact of SGLT2i on severe infection, secondary outcomes included septic shock, sepsis with specific organ dysfunctions (respiratory, cardiovascular, genitourinary, neurological, and hematological systems), and mortality [27]. These outcomes were evaluated to assess the progression of critical illness after the onset of sepsis.

Patient disease progression and outcomes were recorded throughout the study period to obtain a detailed assessment of disease severity after T2DM diagnosis. Therefore, the potential confounding factors included Diabetes Complications Severity Index (DCSI) scores and insulin use after diagnosis of DM were all considered in this study to account for differences in disease severity and treatment history. The DCSI is a validated tool for estimating the severity of complications in DM patients for assessing disease progression [29]. In this study, DCSI scores were calculated based on diagnostic codes during the 12‐month baseline period before the index date.

### 2.4. Statistical Analysis

The baseline characteristics between SGLT2i and DPP‐4i users were compared using standardized mean difference (SMD) to assess the balance of selected variables. An SMD greater than 0.1 was considered to indicate a meaningful imbalance. The incidence trend of sepsis between SGLT2i and DPP‐4i users was illustrated using Kaplan–Meier curves with log‐rank test to compare differences. Moreover, the incidence rate of sepsis was calculated by the number of sepsis divided by the total person‐years of follow‐up and expressed as the number of events per 1,000 person‐years, which was measured from the index date to sepsis, death, or the end of study date. The Cox proportional hazards regression model was used to estimate the risk ratios of sepsis for crude and adjusted hazard ratio (HR) with 95% CI with aggregated sandwich estimator between the SGLT2i users and matched comparison cohorts, DPP‐4i. The multivariable model adjusted for insulin use and DCSI scores since these variables were not included in the initial matching process. The proportional hazards assumption was evaluated using the Schoenfeld residuals test. Further stratified analyses were used to estimate the risk of sepsis across different age, sex, CCI, DCSI, and insulin use groups among DM patients. Additionally, a sensitivity analysis using an as‐treated approach, that patients who had more than 30 days without medication were excluded from follow‐up, was used to evaluate the impact of treatment changes. SAS statistical software Version 9.4 (SAS Institute, Inc., Cary, North Carolina, United States) was used for all analyses. Kaplan–Meier curves were plotted using STATA (Version 12; Stata Corp., College Station, Texas, United States). The statistical significance was set at *p* value <0.05.

## 3. Results

### 3.1. Patients′ Selection

Figure 1 depicts the patient selection process involving adults with diagnosed T2DM during the 2017–2020 period. The initial population included 852,544 individuals with T2DM. After exclusions, the remaining 133,198 patients were divided into two groups: SGLT2i users (*n* = 41,452) and DPP‐4i users (*n* = 91,746). A 1:2 matching process by sex, age, and CCI was then performed, resulting in a final study population of 104,016 patients, consisting of 34,672 SGLT2i users and 69,344 DPP‐4i users.

### 3.2. Clinical Characteristics of Including Patients

Table 1 summarizes the clinical characteristics of the study subjects. After matching by age, sex, and CCI score, SGLT2i users had a lower rate of concomitant insulin use compared with the DPP‐4i group (26.2% vs. 34.8%, SMD = 0.19). Regarding complications, SGLT2i users had a lower DCSI than DPP‐4i users (0.71 ± 1.04 vs. 0.82 ± 1.26, SMD = 0.10).

**Table 1:** The characteristics of diabetes patients between SGLT2i and DPP‐4i users included in the study.

	**Total** **(** **n** = 10, 4016 **)**	**SGLT2i users** **(** **n** = 34,672 **)**	**DPP-4i users** **(** **n** = 69,344 **)**	**SMD**
Sex, *n* (%)				0.00
Male	68,910 (66.2)	22,970 (66.2)	45,940 (66.2)	
Female	35,106 (33.8)	11,702 (33.8)	23,404 (33.8)	
Age group, n (%)				0.00
20–29	2094 (2)	698 (2)	1396 (2)	
30–39	8997 (8.6)	2999 (8.6)	5998 (8.6)	
40–49	22,191 (21.3)	7397 (21.3)	14,794 (21.3)	
50–59	32,925 (31.7)	10,975 (31.7)	21,950 (31.7)	
60–69	27,045 (26)	9015 (26)	18,030 (26)	
70–79	8712 (8.4)	2904 (8.4)	5808 (8.4)	
≥ 80	2052 (2)	684 (2)	1368 (2)	
CCI group, *n* (%)				0.00
0	29040(27.9)	9680(27.9)	19360(27.9)	
1	59787(57.5)	19929(57.5)	39858(57.5)	
2 more	15189(14.6)	5063(14.6)	10126(14.6)	
Insulin	33208(31.9)	9097(26.2)	24111(34.8)	0.19
DCSI, mean ± SD	0.78 ± 1.19	0.71 ± 1.04	0.82 ± 1.26	0.10
DCSI group, *n* (%)				0.10
0	60,634 (58.3)	20,107 (58)	40,527 (58.4)	0.01
1‐2	21,303 (20.5)	7901 (22.8)	13,402 (19.3)	0.09
≥3	22,079 (21.2)	6664 (19.2)	15,415 (22.2)	0.07
Sepsis, *n* (%)	14,238 (13.7)	3488 (10.1)	10,750 (15.5)	0.16
Time to sepsis, median (Q1–Q3)	0.88 (0.33–1.76)	0.87 (0.33–1.69)	0.89 (0.32–1.77)	

### 3.3. Risk of Sepsis

**Figure 2:**
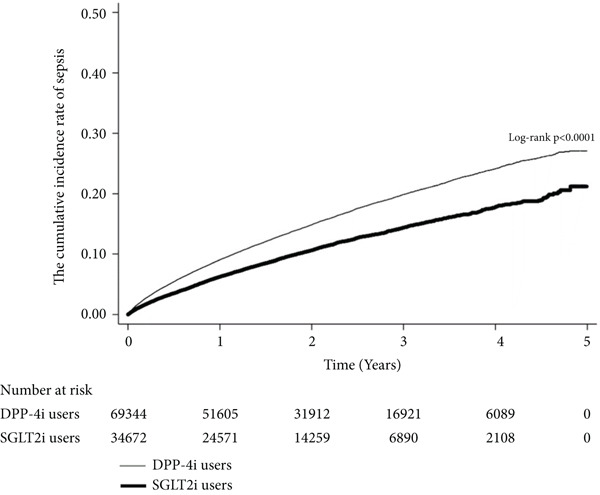
Kaplan–Meier failure curve and cumulative incidence for sepsis.

A Kaplan–Meier curve demonstrated that SGLT2i users had a lower cumulative incidence of sepsis than DPP‐4i users (log‐rank *p* < 0.000) (Figure 2). Table 2 shows the risk of sepsis in diabetes patients between SGLT2i and DPP‐4i users among overall and different subgroups. Compared with DPP‐4i users, SGLT2i users had a lower risk of sepsis (10.1% vs. 15.5%; aHR, 0.77; 95% CI, 0.74–0.81). Additionally, the SGLT2i group had a significantly lower risk of sepsis than the DPP‐4i group across all subgroups according to sex (male: aHR, 0.76 [95% CI: 0.71–0.81]; female: aHR, 0.79 [95% CI: 0.74–0.84]), age (< 65 years: aHR, 0.75 [95% CI: 0.71–0.79]; ≥ 65 years: aHR, 0.84 [95% CI: 0.77–0.93]), CCI (0: aHR, 0.78 [95% CI: 0.72–0.85]; 1: aHR, 0.78 [95% CI: 0.73–0.83]; ≥ 2: aHR, 0.72 [95% CI: 0.64–0.80]), DCSI (0: aHR, 0.82 [95% CI: 0.76–0.89]; 1–2: aHR, 0.82 [95% CI: 0.68–0.98]; ≥ 3: aHR, 0.79 [95% CI: 0.69–0.90]), and concomitant use of insulin (yes: aHR, 0.72 [95% CI: 0.66–0.79]; no: aHR, 0.84 [95% CI: 0.78–0.90]).

**Table 2:** The risk of sepsis in diabetes patients between SGLT2i and DPP‐4i users.

	**SGLT2i users**				**DPP-4i users**				**Crude HR** **(95% CI)**	**aHR** ^**a**^ **(95% CI)**
	**Total**	**Sepsis**	**Person-year**	**Incidence rate** **(per 1000 PY)**	**Total**	**Sepsis**	**Person** **Year**	**Incidence rate** **(per 1000 PY)**		
Overall	34,672	3488 (10.06)	63,629.35	54.82	69,344	10,750 (15.5)	13,8875.68	77.41	0.69 (0.66–0.72)	0.77 (0.74–0.81)
Stratified										
Sex										
Male	22,970	1815 (7.9)	42,833.84	42.37	45,940	5969 (12.99)	93,702.81	63.7	0.66(0.62–0.70)	0.76(0.71–0.81)
Female	11,702	1673 (14.3)	20,795.51	80.45	23,404	4781 (20.43)	45,172.87	105.84	0.73 (0.68–0.78)	0.79 (0.74–0.84)
Age group										
< 65	28,033	2545 (9.08)	53,253.50	47.79	55,474	7660 (13.81)	11,2720.20	67.96	0.68 (0.64–0.71)	0.75 (0.71–0.79)
≥ 65	6639	943(14.2)	10,375.85	90.88	13870	3090 (22.28)	26,155.48	118.14	0.74 (0.68–0.80)	0.84 (0.77–0.93)
CCI group										
0	9680	1058 (10.93)	21,540.27	49.12	19360	3099 (16.01)	45,919.09	67.49	0.70 (0.64–0.75)	0.78 (0.72–0.85)
1	19,929	1822 (9.14)	34,517.47	52.78	39858	5487 (13.77)	75,695.76	72.49	0.72 (0.67–0.76)	0.78 (0.73–0.83)
2 more	5063	608 (12.01)	7571.61	80.3	10126	2164 (21.37)	17,260.83	125.37	0.62 (0.56–0.68)	0.72 (0.64–0.80)
DCSI group										
0	20,107	1539(7.65)	34,601.21	44.48	40,527	4346 (10.72)	77,936.02	55.76	0.78 (0.72–0.85)	0.82 (0.76–0.89)
1–2	7901	806 (10.2)	15,924.90	50.61	13,402	2189 (16.33)	29,104.62	75.21	0.77 (0.65–0.91)	0.82 (0.68–0.98)
≥ 3	6664	1143 (17.15)	13,103.24	87.23	15,415	4215 (27.34)	31,835.04	132.4	0.65 (0.58–0.74)	0.79 (0.69–0.90)
Insulin										
User	9097	1670 (18.36)	17,051.14	97.94	24,111	6477 (26.86)	47,723.84	135.72	0.70 (0.64–0.77)	0.72 (0.66–0.79)
Nonusers	25,575	1818 (7.11)	46,578.21	39.03	45233	4273 (9.45)	91,151.84	46.88	0.84 (0.79–0.91)	0.84 (0.78–0.90)

^a^Adjusted by insulin use and DCSI groups.

### 3.4. Secondary Outcomes

Compared with the DPP‐4i group, the SGLT2i group had a lower risk of septic shock (aHR, 0.70; 95% CI, 0.57–0.87) (Table 3). Regarding sepsis‐related specific organ dysfunction, the SGLT2i group showed a significantly lower risk of respiratory, genitourinary, and neurological dysfunction compared to the DPP‐4i group. Although SGLT2i was also associated with a lower risk of cardiovascular and hematological dysfunction, these differences did not show statistically significance. Lastly, the SGLT2i group could be associated with a lower mortality rate than the DPP‐4i group (aHR, 0.60; 95% CI, 0.54–0.66) (Table 3).

**Table 3:** The risk of secondary outcomes in diabetes patients. (SGLT2i users vs. DPP‐4i users).

	**SGLT2i users** **(** **n** = 34,672 **)**			**DPP-4i users** **(** **n** = 69,344 **)**			**Crude HR** **(95% CI)**	**aHR** ^**a**^ **(95% CI)**
	**Event,** **n** **(%)**	**Person** **Year**	**Incidence rate** **(per 1000 PY)**	**Event, n(%)**	**Person** **Year**	**Incidence rate** **(per 1000 PY)**		
Septic shock	233 (0.67)	68,738.05	3.39	1022 (1.47)	15,5005.82	6.59	0.54 (0.46–0.64)	0.70 (0.57–0.87)
Sepsis‐related organ dysfunction								
Respiratory organ	174 (0.5)	68,793.08	2.53	828 (1.19)	15,5226.37	5.33	0.46 (0.38–0.56)	0.64 (0.49–0.82)
Cardiovascular organ	50 (0.14)	68,873.21	0.73	180 (0.26)	15,5621.82	1.16	0.65 (0.46–0.92)	0.77 (0.46–1.28)
Genitourinary organ	147 (0.42)	68,785.25	2.14	742 (1.07)	15,4906.65	4.79	0.42 (0.34–0.51)	0.52 (0.40–0.68)
Neurological organ	26 (0.07)	68,899.76	0.38	189 (0.27)	15,5631.36	1.21	0.31 (0.20–0.49)	0.45 (0.26–0.79)
Hematological organ	10 (0.03)	68,914.23	0.15	55 (0.08)	15,5724.06	0.35	0.43 (0.21–0.87)	0.59 (0.25–1.36)
Mortality	723 (2.09)	68,929.34	10.49	3490 (5.03)	15,5782.84	22.4	0.48 (0.43–0.52)	0.60 (0.54–0.66)

^a^Adjusted by insulin use and DCSI groups.

### 3.5. Sensitivity Analysis

Table 4 presented the sensitivity analysis using an as‐treated approach among SGLT2i and DPP‐4i users. The results remained consistent with the primary analysis. Use of the SGLT2i group was associated with a significantly lower risk of sepsis compared with the DPP‐4i (aHR, 0.73; 95% CI, 0.67–0.80). Similar protective effects were observed for organ dysfunction, including the respiratory (aHR, 0.65; 95% CI, 0.43–0.98), genitourinary (aHR, 0.48; 95% CI, 0.29–0.78), and neurological systems (aHR, 0.17; 95% CI, 0.04–0.72). The risk of mortality was also significantly lower in the SGLT2i group (aHR, 0.66; 95% CI, 0.54–0.80) compared with that of the DPP‐4i group.

**Table 4:** Sensitivity analysis using an as‐treated approach among SGLT2i and DPP‐4i users.

	**SGLT2i users** **(** **n** = 11071 **)**			**DPP-4i users** **(** **n** = 19953 **)**			**Crude HR** **(95% CI)**	**aHR** ^**a**^ **(95% CI)**
	**Event,** **n** **(%)**	**Person-year**	**Incidence rate** **(per 1000 PY)**	**Event,** **n** **(%)**	**Person-year**	**Incidence rate** **(per 1000 PY)**		
Sepsis	646 (5.84)	25,975.74	24.87	1885 (9.64)	47,678.14	39.54	0.64 (0.59–0.70)	0.73 (0.67–0.80)
Septic shock	39 (0.35)	26,691.71	1.46	150 (0.75)	49,762.20	3.01	0.51 (0.36‐0.72)	0.73 (0.51–1.04)
Sepsis‐related organ dysfunction								
Respiratory organ	28 (0.25)	26,703.08	1.05	122 (0.61)	49,780.13	2.45	0.45 (0.30–0.68)	0.65 (0.43–0.98)
Cardiovascular organ	5 (0.05)	26,714.12	0.19	26 (0.13)	49,824.27	0.52	0.37 (0.14–0.96)	0.55 (0.21–1.44)
Genitourinary organ	19 (0.17)	26,708.44	0.71	109 (0.55)	49,744.02	2.19	0.34 (0.21–0.56)	0.48 (0.29–0.78)
Neurological organ	2 (0.02)	26,718.69	0.07	28 (0.14)	49,830.87	0.56	0.13 (0.03‐0.56)	0.17 (0.04–0.72)
Hematological organ	0 (0)	26,719.07	0.00	2 (0.01)	49,843.12	0.04	NA	NA
Mortality	129 (1.17)	26,719.07	4.83	517 (2.59)	49,844.44	10.37	0.49 (0.40–0.59)	0.66 (0.54–0.80)

^a^Adjusted by sex, age groups, CCI groups, insulin use, and DCSI groups.

## 4. Discussion

This study compared the effect of SGLT2i versus DPP‐4i on the risk of sepsis among patients with DM. We found that the use of SGLT2i was associated with a 23% reduced risk of sepsis compared to DPP‐4i. These results remained consistent across all prespecified subgroups, including sex, age, comorbidities, severity of DM, and concomitant insulin use. Additionally, SGLT2i was associated with a lower risk of sepsis, septic shock, and various organ dysfunction. Overall, our findings suggest a potential benefit of SGLT2i in reducing the risk of sepsis among patients with DM.

Our results align with previous studies that have shown an association between SGLT2i use and reduced infection risk [19, 20, 30]. A meta‐analysis of randomized controlled trials (RCTs) reported that, compared with placebo, SGLT2i use was linked to a reduced risk of pneumonia (RR, 0.87; 95% CI, 0.78–0.98) and septic shock (RR, 0.65; 95% CI, 0.44–0.95) in patients with DM [19]. However, there may be reporting and ascertainment biases, as pneumonia and septic shock were not prespecified outcomes in the included RCTs, and the definitions of these outcomes varied across studies [19]. A retrospective cohort study using Taiwan’s claims database also reported that patients receiving SGLT2i had a lower risk of sepsis/septic shock than nonusers (aHR, 0.63; 95% CI, 0.59–0.66) [20]. In contrast to these two studies [19, 20], our study employed an active comparator (DPP‐4i) design. As both SGLT2is and DPP‐4is are newer classes of oral antidiabetic agents, they have increasingly become integral components of T2DM management, particularly in patients who require additional glycemic control beyond metformin or have comorbidities such as cardiovascular or renal disease. These two classes are frequently prescribed at similar stages of disease progression and are often considered interchangeable options in routine clinical practice [22]. By comparing SGLT2i to an established therapy like DPP‐4i rather than a placebo, we provide more clinically relevant information for healthcare decision‐making. Similarly, a retrospective study using a territory‐wide clinical registry in Hong Kong found that, compared with DPP‐4i, SGLT2i could be associated with a lower risk of pneumonia (aHR 0.63; 95% CI, 0.55–0.72) and sepsis (aHR 0.52; 95% CI, 0.44–0.62) [30]. Consistent with our findings, this suggests that SGLT2i may be superior to DPP‐4i in preventing sepsis. Although both SGLT2i and DPP‐4i are newer oral antidiabetic drugs typically prescribed to patients at similar stages of T2DM [22], SGLT2i may be the better option, given the increased vulnerability of patients with DM to sepsis.

Although this study showed an association between SGLT2i and sepsis, the mechanism by which SGLT2i might lower the risk of sepsis remains unclear. Possible mechanisms include cardiac [16] and renal [31–33] protection, which may directly or indirectly reduce the risk of sepsis. In addition, SGLT2i may exhibit immune modulation through its anti‐inflammatory activity [34, 35]. A previous study [33] indicated that SGLT2i lowers levels of inflammatory cytokines like interleukin‐6, matrix metalloproteinase‐7, and high‐sensitivity C‐reactive protein in diabetic patients, potentially preventing focal infections from progressing to sepsis or septic shock. SGLT2i also helps promote endogenous endothelial repair via activation of AMPK‐mediated inhibition of inflammation [36], independent of glucose‐lowering effects, addressing widespread endothelial dysfunction—a major factor in sepsis‐related mortality [37]. However, further study is warranted to investigate the potential mechanisms.

Besides the primary outcomes, our study demonstrated survival benefits comparing SGLT2i with DPP‐4i. We found that SGLT2i was associated with a lower mortality rate than DPP‐4i (aHR, 0.60; 95% CI, 0.54–0.66). Our findings are consistent with previous network meta‐analysis [38], in which SGLT2i was associated with significantly lower all‐cause mortality compared with DPP‐4i. Specifically, SGLT2i showed a HR of 0.78 (95% CI, 0.68–0.90), which translates to an absolute risk difference of −0.9% (95% CCI, −1.2% to −0.4%) when compared with DPP‐4i [38]. Furthermore, the same benefit was also found in an Italian real‐world study [39], showing that patients with T2DM who initiated SGLT2i had lower all‐cause mortality than those who initiated DPP‐4i. However, further RCT is warranted.

Although prior studies have examined the association between SGLT2i use and infection risk, most studies focused on overall sepsis. Our study has several strengths to address this knowledge gap by investigating severe infection outcomes using a large nationwide cohort. First, a large nationwide claims database covering the whole Taiwanese population under a single‐payer health insurance system was used in this study. Therefore, more study subjects were included, and the findings could also be compared across different healthcare settings. Second, we extended the scope of outcomes beyond overall sepsis to include specific organ dysfunctions (respiratory, cardiovascular, genitourinary, neurological, and hematological systems) and all‐cause mortality to further assess more comprehensive impacts of SGLT2i on severe infection‐related consequences. Finally, by demonstrating consistent associations across multiple severe infection outcomes in a large population, our results improve the generalizability of the potential protective effects of SGLT2i and provide additional evidence to assist clinical decision‐making.

This study had several limitations. First, although we observed that the all‐cause mortality risk in the SGLT‐2i group was better than that in the DPP‐4i group, we could not determine the specific cause of mortality. It is unclear whether the improvement in sepsis risk contributed to the decreased mortality, or if it was due to the intrinsic cardiac and renal benefits of SGLT‐2i. Thus, future studies could include the cause‐specific mortality information to clarify these relationships. Second, the potential misclassification bias may have existed on administrative databases, particularly regarding diagnosis coding, which could affect results. However, previous studies have validated the coding accuracy of the NHIRD, and certification undergoes expert review to ensure a high level of diagnostic precision. Therefore, the potential for misclassification bias is minimized. Third, differences in clinical management and treatment adherence between SGLT2i and DPP‐4i users may remain unaccounted for, potentially impacting the generalizability of the findings. To further evaluate the impact of treatment changes, we presented a sensitivity analysis using an as‐treated approach. Only patients who continuously used their assigned drug without treatment gaps more than 30 days were included in this sensitivity analysis. The results support our findings and reduce concerns about exposure misclassification. Forth, data on the concurrent or prior use of other non‐insulin antidiabetic drugs (NIADs) were not included in the analysis. This may limit our finding to estimate the effects of SGLT2i or DPP‐4i. Future research should consider the influence of concomitant antidiabetic therapies. For reducing the potential confounding bias, we used exact matching on age, sex, and CCI score, and the estimated model also adjusted for DCSI score and insulin use. However, concurrent use of other medications could not be fully considered. Although DCSI and CCI partly reflect cardiovascular comorbidities, specific medication exposures, such as antithrombotic therapies, were not included to avoid potential bias from drug–drug interaction. Therefore, residual confounding related to unmeasured concomitant treatments cannot be excluded, and the findings should be interpreted with caution. Lastly, we were unable to assess the duration of diabetes prior to cohort entry or measures of glycemic control, such as time‐weighted HbA1c, due to the absence of laboratory data in the claims database. Especially, the calculated *E*‐values were 1.69 for sepsis and 1.88 for septic shock, indicating the unmeasured confounding factors should be considered. Although the sensitivity analysis presented consistent results, residual confounding cannot be completely excluded. Therefore, the findings should be carefully interpreted.

## 5. Conclusion

This study demonstrates that the use of SGLT2i could be associated with a significantly reduced risk of sepsis compared with DPP‐4i in patients with T2DM. The findings were consistent across various subgroups and indicated a lower risk of severe sepsis, septic shock, and site‐specific infections, as well as reduced mortality. These results align with previous evidence suggesting the potential of SGLT2i to lower infection risk and prevent the progression of infections to life‐threatening stages in patients with diabetes. Given these advantages, SGLT2i may be a superior therapeutic option over DPP‐4i for reducing the risk of sepsis in T2DM patients, further supporting their role in the comprehensive management of diabetes. However, further studies are needed to fully understand the underlying mechanisms and to confirm these findings in broader, real‐world populations.

## Ethics Statement

This study was conducted in accordance with the guidelines of the Declaration of Helsinki and was approved by the Research Ethics Committee of Chi Mei Hospital (IRB:11301‐012, January 22, 2024).

## Disclosure

The authors have nothing to report.

## Conflicts of Interest

The authors declare no conflicts of interest.

## Author Contributions

Chun‐Ying Wu: Conceptualization and writing—original draft. Chih‐Cheng Lai: Conceptualization, writing—review and editing, and validation. Chung‐Han Ho: Writing—original draft, formal analysis, and funding acquisition. Yu‐Cih Wu: Formal analysis, and visualization. Kuang‐Ming Liao: Resources, and data curation. Jhi‐Joung Wang: Conceptualization, supervision, and methodology. Ding‐Chau Wang: Resources and methodology. Fu‐Wen Liang: Methodology, writing—review and editing, formal analysis, and validation. Chun‐Ying Wu and Chung‐Han Ho contributed equally to this work.

## Funding

This work was supported by Chi Mei Medical Center (10.13039/501100006578), CMFHR113029.

## Supporting information


**Supporting Information** Additional supporting information can be found online in the Supporting Information section. **Appendix A** (Table S1: International Classification of Diseases, 10th Revision, Clinical Modification (ICD‐10‐CM) Diagnosis for outcomes; Figure S1 Kaplan–Meier failure curve and cumulative incidence for septic shock).

## Data Availability

The data sources are the Taiwan Nation Health Insurance Database and Taiwan Cancer Registry. The data are available with permission from the Taiwan Health and Welfare Data Science Center (https://dep.mohw.gov.tw/DOS/cp-5119-59201-113.html, accessed on 07 November 2024). Restrictions apply to the availability of these data, which were used under license for this study.
