# Correction to “Association Between Sodium–Glucose Cotransporter‐2 Inhibitors and Sepsis Risk in Patients With Type 2 Diabetes Mellitus”

**DOI:** 10.1155/jdr/9805928

**Published:** 2026-04-30

C. Y. Wu, C. C. Lai, C. H. Ho, et al., “Association Between Sodium‐Glucose Cotransporter‐2 Inhibitors and Sepsis Risk in Patients With Type 2 Diabetes Mellitus,” *Journal of Diabetes Research*, 2026 (2026): 1437417, https://doi.org/10.1155/jdr/1437417.

In the published article, the funding information was incomplete. The Funding section should have read: “This work was supported by Chi Mei Medical Center, CMFHR113029 and National Science Technology Council, NSTC 114‐2314‐B‐037‐029.” This correction relates only to the funding information and does not affect the scientific content, results, or conclusions of the article.

We apologize for this error.

