# Supplementary material for: Association Between Sodium–Glucose Cotransporter‐2 Inhibitors and Sepsis Risk in Patients With Type 2 Diabetes Mellitus
Source: J Diabetes Res. 2026 Jan 8;2026:1437417. doi: 10.1155/jdr/1437417 (PMC12782002; doi:10.1155/jdr/1437417)
Supplement: Supplementary file 1 — Supporting Information Additional supporting information can be found online in the Supporting Information section. Appendix A (Table S1: International Classification of Diseases, 10th Revision, Clinical Modification (ICD‐10‐CM) Diagnosis for outcomes; Figure S1 Kaplan–Meier failure curve and cumulative incidence for septic shock). [file JDR-2026-1437417-s001.zip › Supplemental Figure.pptx]

## Slide 1
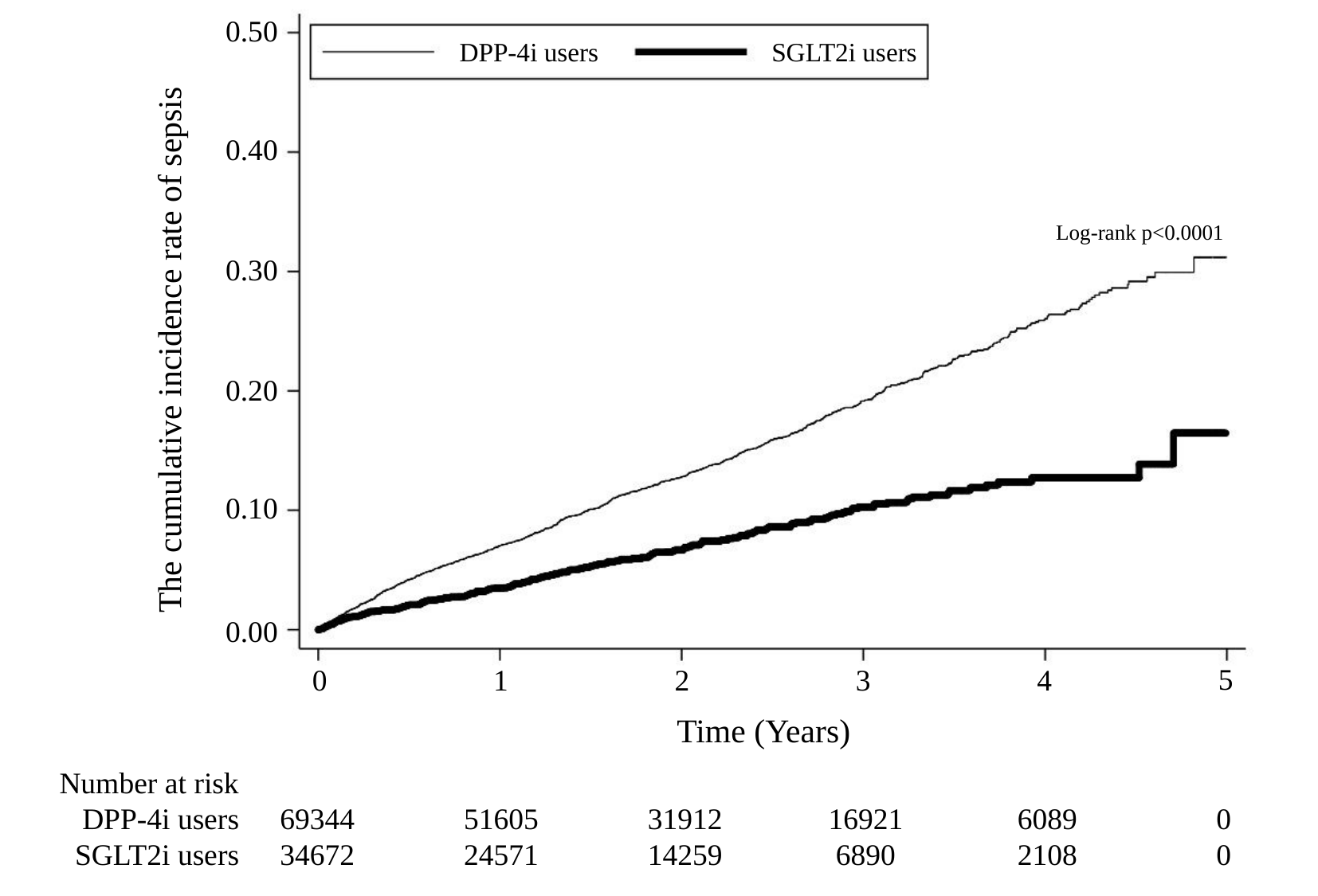

0.50
0.40
0.30
0.20
0.10
0.00
DPP-4i users
SGLT2i users
Log-rank p<0.0001
The cumulative incidence rate of sepsis
5
1
2
3
4
0
Time (Years)
Number at risk
DPP-4i users
SGLT2i users
69344
34672
51605
24571
31912
14259
16921
6890
6089
2108
0
0
