# Supplementary material for: Association Between Sodium–Glucose Cotransporter‐2 Inhibitors and Sepsis Risk in Patients With Type 2 Diabetes Mellitus
Source: J Diabetes Res. 2026 Jan 8;2026:1437417. doi: 10.1155/jdr/1437417 (PMC12782002; doi:10.1155/jdr/1437417)

**Supplemental table .** **International Classification of Diseases, Ninth Revision, Clinical Modification (ICD-10-CM) Diagnosis for outcomes.**

| **Variable** | **ICD-10-CM** |
| --- | --- |
| Type 2 DM | E11 |
| **Outcome** |  |
| Sepsis | A03.9, A02.1, A04.7,A20.7, A21.7, A22.7, A23.9, A24.1, A26.7, A28.0, A28.2, A32.7, A39.2, A39.3,A39.4, A40, A41, A42.7, B00.7, B37.7, B95.48, B95.6, B96.2, J18.9, J44.0, N39.0, P36.0, P36.1, P36.2, P36.3, P36.4, P36.5, P36.8, P36.9, P35.2, P37.2, P37.5 |
| Sepsis without septic shock | R65.20 |
| Septic shock | R57.2, R65.21 |
| Sepsis related organ dysfunction | |
| Respiratory | J96.0, J96.9, J80, R09.2 |
| Cardiovascular | R57.0, R57.1, R57.2, R57.8, R57.9, I95.1, I95.9 |
| Renal | N17.0, N17.1, N17.2, N17.8, N17.9 |
| Neurological | K72.0, K72.9, K76.3, F05.0, F05.9, G93.1, G93.4, G93.80 |
| Haematological | D69.5, D69.6, D65 |
| **Drug** | **ATC code** |
| SGLT2i | A10BK* |
| DPP-4i | A10BH* |

**Supplemental Figure.** Kaplan-Meier Failure Curve and Cumulative Incidence for septic shock


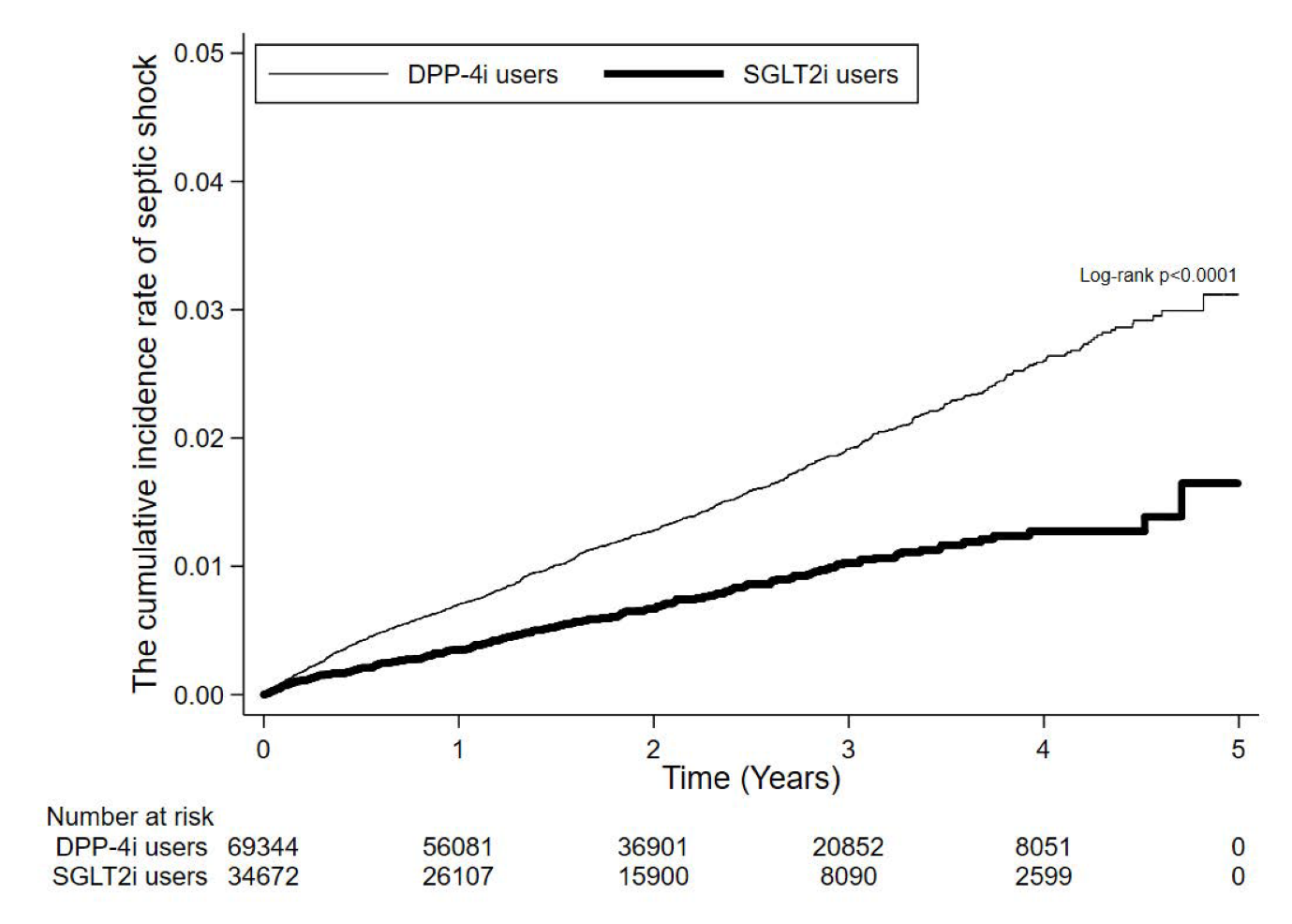

Supplement: Supplementary file 1 — Supporting Information Additional supporting information can be found online in the Supporting Information section. Appendix A (Table S1: International Classification of Diseases, 10th Revision, Clinical Modification (ICD‐10‐CM) Diagnosis for outcomes; Figure S1 Kaplan–Meier failure curve and cumulative incidence for septic shock). [file JDR-2026-1437417-s001.zip › Supplementary.docx]
